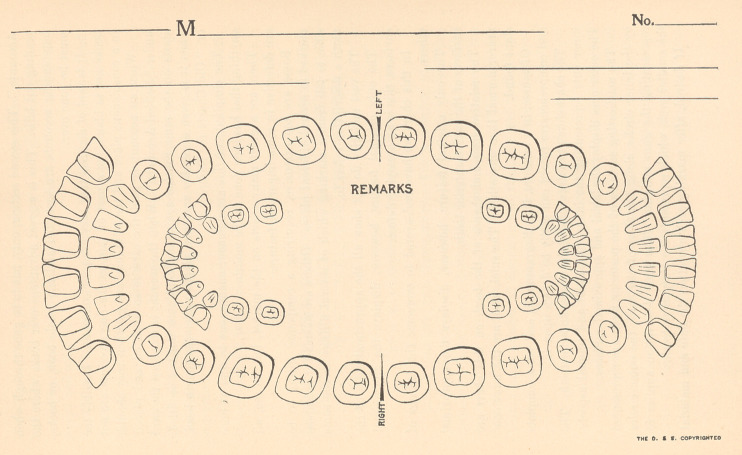# A Card System for Keeping Dental Records and Accounts

**Published:** 1904-01

**Authors:** Walter E. Decker

**Affiliations:** Boston, Mass.


### A CARD SYSTEM FOR KEEPING DENTAL RECORDS AND ACCOUNTS.[1]

BY WALTER E. DECKER, BOSTON, MASS.

System is an assemblage of objects in regular arrangement.

Index is that which guides, points out, informs, or manifests. An index system, therefore, as applied in our present subject, is a regular arrangement of cards that clearly point out or inform.

The value of careful records and accounts cannot be too greatly emphasized.

The conditions that exist during the treatment of a tooth at one time may very materially affect the course pursued later. The observation of different methods and materials under known conditions is a teacher that no successful man can possibly do without.

A systematic record requires very little time, frequently, not more than one minute for each operation.

A dentist's time is his capital, and he can ill afford to waste valuable minutes when they can easily be saved. Furthermore, the loss of energy must be considered. Thought is energy expended, and anything that can be done with little thought and effort conserves energy. A search through several books and over many pages for a particular record is not only a waste of time and energy, but a sore aggravation. Record ledgers, if used as much as they should be, soon wear out and must be rebound and new indices written, or a new book added to the list.

For these reasons a card system is particularly adapted to dentists. The principal arguments in its favor can be clearly stated under the following heads: Adaptability, Contractibility, Expansibilty, and Accessibility.

Its adaptability to dentists is its most conspicuous feature. In few callings are there occasions to refer to past records as frequently as in our profession, and at such times there are few men whose minutes are as valuable.

With books it is necessary to refer, first, to the index, then to the page, or, likely, to several pages, and, finally, to the record. Then, too, it is not convenient to have the books at the chair during an examination, for instance, while the cards may lie upon the

[1] Read before the Massachusetts Dental Society, June 3 and 4, 1903.

tray and the record be referred to without even taking the mirror from the patient's mouth. As I have said, this sort of observation and comparison is a most valuable teacher. Our methods change from time to time, likewise our materials. If we may know by a glance the method or material employed at a previous sitting, it may be of great value in choosing and eliminating.

A card system has contractibility,—no useless matter need be retained. In the book old matter which has ceased to be of use continues to occupy valuable space, and there is no way of weeding it out except by rewriting. In the card index active and dead matter may be separated and yet one class referred to as readily as the other. The card containing the dead matter may be removed without interfering with the remaining cards in any way. As a result the card list is always an accurate up-to-date source of information.

The expansibility of a card system is limitless.

Additions can be made by the insertion of new cards without disturbing the former occupants.

It is far less expensive than keeping books. One outfit answering the purpose of any number of new ledgers. The original outlay is only once. After that one only buys the leaves, as it were.

It also has accessibility,—always "get-at-able." For convenience of reference nothing can equal the card index. The record cards are arranged under certain heads and subdivided under alphabetical guides in such manner that from the patient's name his card can be turned to instantly and, as I will show you later, when the card is in one's hand the record stands out clear and distinct.

Not only do I advise the casting aside of record books, but also other books, papers, etc., whose matter can be systematically recorded in the same cabinet. For instance, a cash account, a directory, prescriptions under certain heads, various filling-materials and medicaments, legal and other matters of whatever nature can be indexed in very convenient forms.

With a record pertaining to the practice, four divisions, each with alphabetical guides, is the most convenient arrangement. One for "Unfinished Operations," one for "Outstanding Accounts," one for "Settled Accounts," and one for "Old Charts for Reference." During a series of sittings for a patient the card is kept under "Unfinished Operations." When the mouth has been placed in order at the last sitting the card is placed under "Outstanding

M ______________________ No. ________

THE D. & S. COPYRIGHTED

Accounts." This second section, only, has to be gone through when sending out bills. This is a great advantage over books where the outstanding accounts are scattered and the whole book, or books, must be gone over.

It is said of dentists that they are poor collectors. Possibly the books are to blame for a part of this, as the due accounts are not in a conspicuous place and the bills may not go when they should.

When an account is paid the card is so credited and placed under "Settled Accounts." When the account is reopened the card starts on the same routine again and continues until it is filled, when it is given a number and then placed under the fourth head, or, "Old Charts for Reference."

The simplicity of this arrangement enables one to find at once anything that has been done in the mouth.

There are a great many charts on the market, but, to me, all are defective in some important feature.

Some are too extensive, requiring a compound filling to be marked in several different places. Others are so small it is impossible to tell from a marking which of several fillings in the same tooth a description refers to.

The vast majority do not show all the approximal sides of the

The only original feature, however, displays the approximal surfaces, this is an annoying defect.

In this chart we have endeavored to overcome the failings of others.

The only original feature, however, displays the approximal surfaces of the twelve anterior teeth.

The relative sizes and shapes of the teeth on this chart are nearer normal than any other. The sulci, also, are the most constant found.

Of course, in a measure, it is diagrammatic, and yet a complete and comprehensive record can easily be drawn upon it.

The chart shows two drawings of the twelve anterior teeth. The inner cuts representing the inner, or palatal and lingual aspects. The outer ones display the labial and approximal surfaces.

The masticating surfaces of the posterior teeth are represented by the inner circles along the sides of the card, the outer circles representing the gum margins. The same description applies to the smaller arch, which represents the temporary teeth.

If desired, after marking each operation the date and material used can be written opposite, and only the charge placed upon the back of the card. The better way, however, is to give each operation a number, which is also written on the back of the card with the record and account.

The back is ruled in such a manner that the date, operation, and charge can be recorded thereon. As there are fifty lines, one card will last a long time.

These cards can be obtained of the Eugene Smith Company, 108 Pemberton Building, Makers of Special Card Index Systems for Physicians and Dentists. Mr. Smith has kindly consented to keep this card in stock and arrange outfits in any style and for almost any price.

The popular outfit costs seven dollars, and contains cards enough to last from six to ten years.

## Figures and Tables

**Figure f1:**